# Observer variability of reference tissue selection for relativecerebral blood volume measurements in glioma patients

**DOI:** 10.1007/s00330-018-5353-y

**Published:** 2018-03-23

**Authors:** Marcel T. H. Oei, Frederick J. A. Meijer, Jan-Jurre Mordang, Ewoud J. Smit, Albert J. S. Idema, Bozena M. Goraj, Hendrik O. A. Laue, Mathias Prokop, Rashindra Manniesing

**Affiliations:** 1 Department of Radiology and Nuclear Medicine, Radboudumc, Geert Grooteplein 10, P.O. Box 9101, 6500 HB Nijmegen, The Netherlands; 2 Department of Neurosurgery, Radboudumc, Geert Grooteplein 10, P.O. Box 9101, 6500 HB Nijmegen, The Netherlands; 3 Fraunhofer MEVIS, Universitätsallee 29, 28359 Bremen, Germany

**Keywords:** Cerebral blood volume, Observer variation, Perfusion imaging, Magnetic resonance angiography, Glioma

## Abstract

**Objectives:**

To assess observer variability of different reference tissues used for relative CBV (rCBV) measurements in DSC-MRI of glioma patients.

**Methods:**

In this retrospective study, three observers measured rCBV in DSC-MR images of 44 glioma patients on two occasions. rCBV is calculated by the CBV in the tumour hotspot/the CBV of a reference tissue at the contralateral side for normalization. One observer annotated the tumour hotspot that was kept constant for all measurements. All observers annotated eight reference tissues of normal white and grey matter. Observer variability was evaluated using the intraclass correlation coefficient (ICC), coefficient of variation (CV) and Bland-Altman analyses.

**Results:**

For intra-observer, the ICC ranged from 0.50–0.97 (fair–excellent) for all reference tissues. The CV ranged from 5.1–22.1 % for all reference tissues and observers. For inter-observer, the ICC for all pairwise observer combinations ranged from 0.44–0.92 (poor–excellent). The CV ranged from 8.1–31.1 %. Centrum semiovale was the only reference tissue that showed excellent intra- and inter-observer agreement (ICC>0.85) and lowest CVs (<12.5 %). Bland-Altman analyses showed that mean differences for centrum semiovale were close to zero.

**Conclusion:**

Selecting contralateral centrum semiovale as reference tissue for rCBV provides the lowest observer variability.

**Key Points:**

*• Reference tissue selection for rCBV measurements adds variability to rCBV measurements.*

*• rCBV measurements vary depending on the choice of reference tissue.*

*• Observer variability of reference tissue selection varies between poor and excellent.*

*• Centrum semiovale as reference tissue for rCBV provides the lowest observer variability.*

## Introduction

T2*-weighted dynamic susceptibility contrast-enhanced MR imaging (DSC-MRI) has been shown to be useful in evaluating brain neoplasms. With DSC-MRI, T2*-weighted echo planar images are acquired that measure the signal intensity change over time after the injection of a bolus of a paramagnetic contrast agent. The change in relaxation rate (ΔR2*) can be calculated from the signal intensity and is proportional to the contrast agent in the tissue. Cerebral blood volume (CBV) is a parameter that can be measured with DSC-MRI, and is proportional to the area under the curve of ΔR2*(t). Studies have used CBV to differentiate between low- and high-grade gliomas [1, 2], to differentiate tumour progression from pseudo-progression [3], and to assess treatment response to anti-angiogenic drug therapy [4–6].

CBV measurements may show high variability. Values vary due to different image acquisition protocols and post-processing methods and also due to physiological differences in the patients such as cardiac output and haematocrit values. This makes it difficult to compare CBV among patients and studies. Therefore, CBV is typically normalized to a reference tissue. The relative CBV (rCBV) is calculated by the CBV in the region of interest (e.g. tumour hotspot) divided by the CBV of an internal reference tissue for normalization. A typical reference tissue is the contralateral normal-appearing white matter (NAWM) or the normal-appearing grey matter (NAGM).

Different reference tissues are used in the literature, including normal-appearing white or grey matter (contralateral or ipsilateral) [7], the contralateral NAWM [2, 8], the contralateral NAGM [9], contralateral thalamus [10] or contralateral centrum semiovale [11]. Most studies do not describe their exact location or give an exact definition, but it is known for instance that CBV values of grey matter are higher than CBV values of white matter [12].

The rCBV is subject to observer variability when the regions of interests (ROIs) are manually annotated. Variability can be reduced by the use of (semi-)automated methods [13–15], but despite their existence, they are not commonly available, and manual annotations of the reference tissue by an experienced radiologist is still common practice [16, 17] for which a reliable and reproducible reference ROI is necessary. Wetzel et al. [18] investigated the observer variability of annotating the tumour hotspot while keeping the internal reference tissue constant. The authors did not study the observer variability of annotating the reference tissue.

Thus, the purpose of this study was to assess the observer variability of rCBV measurements depending on the choice of the reference tissue that is used for normalization in DSC-MRI of glioma patients.

## Materials and methods

### Patient selection

For this retrospective study informed consent was waived. Between 2006 and 2008 our institution participated in a European project called eTumour. Patients presenting with symptoms suggestive of brain tumour, newly diagnosed and untreated brain tumours were prospectively included. One day before surgery they underwent conventional MR, MR spectroscopy and MR perfusion (DSC-MRI) imaging. Our institution included 98 subjects with brain tumours. Further details of the eTumour project can be found in Julia-Sape et al. [19].

The patient selection criteria to select patients from the eTumour database were as follows: (1) Subjects with a histopathologically confirmed diagnosis of glioma was available; (2) subjects did not have surgical resection, biopsy or radiation therapy before DSC-MR imaging was performed; and (3) subjects were 18 years or older. Subjects were excluded if the DSC-MRI was technically inadequate due to motion and susceptibility artifacts.

In total 44 consecutive patients (17 female; 27 male; median age, 58 years; range, 21–79 years) were included. The patients were diagnosed with pathologically proven gliomas (one subependymal giantcell astrocytoma, five low-grade astrocytomas, two low-grade oligoastrocytomas, one anaplastic astrocytoma, five anaplastic oligodendrogliomas and 30 glioblastoma multiforme).

All tumours were located supratentorially.

## MR imaging

MR sequences were acquired on a 3T system (MAGNETOM Trio; Siemens, Erlangen, Germany). The acquisition protocol included an axial T2-weighted (T2w) sequence (repetition time and echo time TR/TE = 4,040/102 ms) or an axial transverse fluid-attenuated inversion recovery (FLAIR) sequence (TR/TE 13,050/103 ms) and an axial T1-weighted (T1w) spin-echo sequence (TR/TE 2,300/4.7 ms) performed before and after intravenous administration of gadoteratemeglumine (Dotarem; Guerbet, Paris, France). The axial contrast-enhanced T1w spin-echo sequence was performed after acquisition of DSC-MRI.

DSC-MRI was performed with a gradient-echo echo-planar imaging sequence (GE-EPI) during the first pass of a (0.1 mmol/kg) bolus of gadoteratemeglumine at a rate of 2.5 ml/s. Imaging parameters were as follows: TR/TE 1,670/45 ms; FOV 230×230 mm; matrix 128×128; voxel size 1.8×1.8×5.0 mm^3^; intersection gap 30 %; flip angle 90°; signal bandwidth 1,346 Hz/x. Fifteen axial sections were obtained through the brain.

## Observers

One observer (MTHO, with 3 years of experience) annotated the tumour hotspot according to the method described by Wetzel et al. [18] and did not participate in annotating the reference tissues.

Three observers (FJAM and BMG, certified neuroradiologists with 10 and 30 years of experience, respectively, and EJS, a resident radiology) independently performed all measurements for the reference tissues. The observers were blinded to patient history and diagnosis. The observers underwent a training session with five training cases, which were excluded in the final evaluation in order to limit performance bias.

## Image analysis

The DSC-MR images were processed on a dedicated in-house developed workstation (Cirrus Brain MR, version 7335, Radboudumc, Nijmegen, The Netherlands). Processing consisted of image registration of DSC-MRI to the T1w image, followed by calculation of the CBV perfusion map [20]. The Weisskoff correction method was used to correct for T1 leakage effects [21, 22]. The DSC-MR image, corresponding CBV map and conventional MR images (T1w before and after contrast, T2w or FLAIR) were made available in the workstation to the observers.

One observer (MTHO) defined the tumour hotspot following Wetzel et al. [18] by annotating four to six circular ROIs of 25 mm^2^ in the area of the tumour hotspot and selecting the ROI with the highest CBV value. This region was then kept constant for all observers in the subsequent normalization step. Defining the tumour hotspot was done in a separate session and care was taken to avoid areas of necrosis, cysts, or non-tumour macro-vessels.

To evaluate the influence normalization for calculating rCBV, all observers were asked to place a reference ROI in a homogenous region at the contralateral side of approximately 25 mm^2^ in the:

- NAWM on the axial section deemed most appropriate by the observers
- NAGM on the axial section deemed most appropriate by the observers
- NAWM on the same axial section as the tumour ROI
- NAGM in the putamen
- frontal NAWM
- parietal NAWM
- NAGM in the thalamus
- centrum semiovale.

**Fig. 1:**
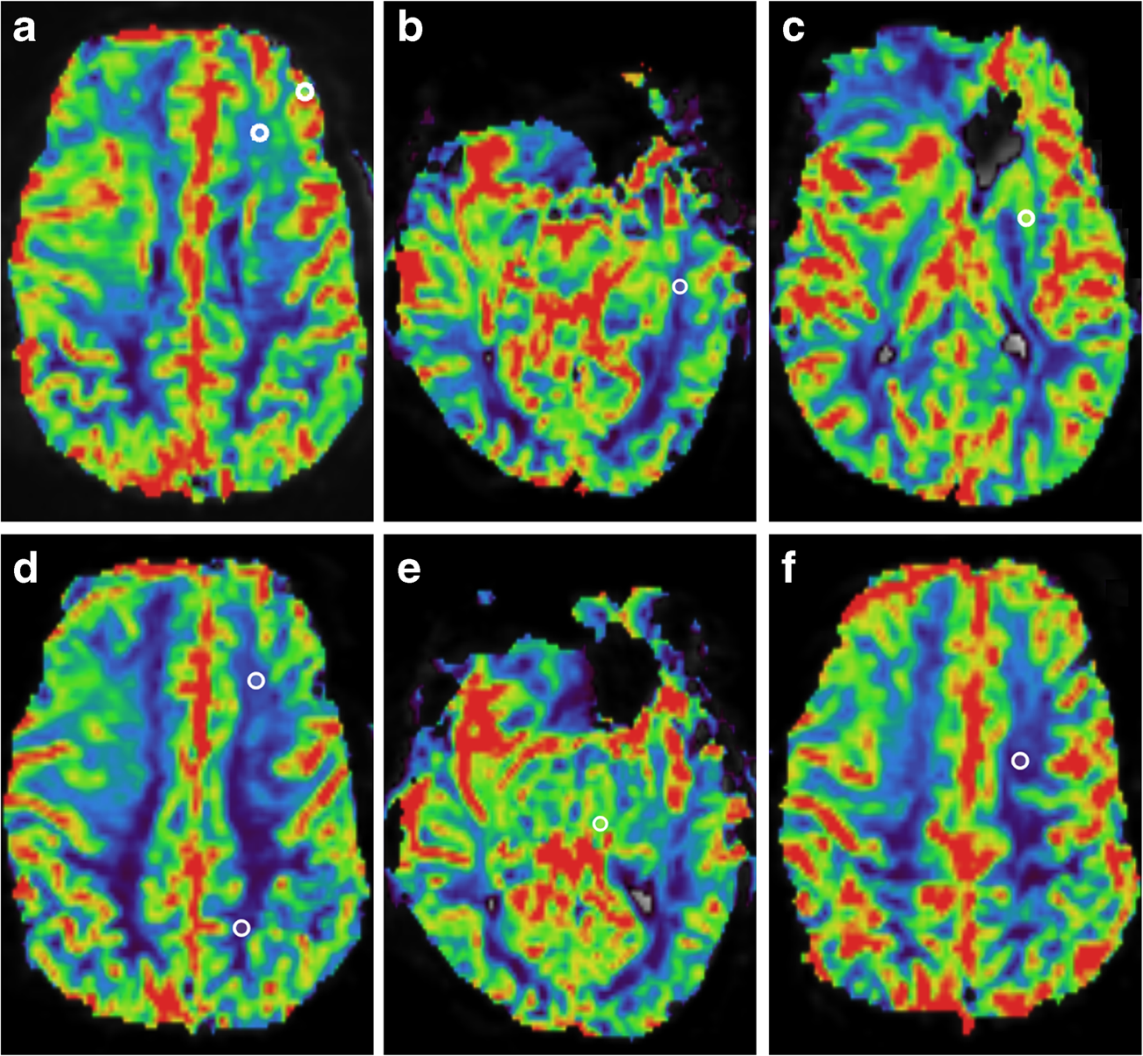
Examples of region of interest (ROI) placement in a patient with a glioblastoma multiforme in the temporal lobe in the right hemisphere. ROI placement in the contralateral hemisphere (left) in the eight regions: (A) Normal-appearing white matter (NAWM) by choice and grey matter (GM) by choice. (B) NAWM in a slice of tumour hotspot. (C) Putamen. (D) frontal and parietal NAWM. (E) Thalamus. (F) Centrum semiovale

Large vessels and tumour-suspicious regions were avoided. rCBV was calculated by dividing the tumour CBV by the CBV of the reference tissue. Figure 1 shows an example of ROI placement in the regions listed above.

## Statistical analysis

Statistical analyses were performed with IBM SPSS Statistics version 20 (SPSS Inc., Chicago, IL, USA). The mean and standard deviation of the rCBV measurements were determined per reference tissue, evaluation session and observer. Normal distribution was tested using the Shapiro-Wilk test. A paired *t*-test was used to compare the rCBV measurements between the two evaluations for every observer and all reference tissues, with *p*<0.05 considered statistically significant.

For the statistical analyses of the observer variability, the coefficient of variation (CV), intraclass correlation coefficient (ICC) and Bland-Altman analyses were used. The CV was calculated for the rCBV for every reference tissue, and every observer. The ICC is reported with a 95 % confidence interval where ICC <0.4 was considered poor agreement, ICC 0.40–0.59 was considered fair agreement, ICC 0.60–0.74 was considered good agreement, and ICC >0.74 was considered excellent agreement [23]. Bland-Altman analyses were expressed as the mean difference, standard deviation and 95 % limits of agreement.

## Intra-observer variability

The observers repeated the rCBV measurements after 2 weeks or longer (up to a month) to assess the intra-observer variability. The measurements were made on the same dataset but in a different random order of presentation to limit recall bias. The statistical analyses were calculated for rCBV measurements between the two evaluations of an observer, for each reference tissue. A two-way mixed ICC model, with absolute agreement, single measures and a 95 % confidence interval was used.

## Inter-observer variability

The statistical analyses were calculated for every pairwise combination of observers to assess the inter-observer variability. Only the rCBV measurements of the first evaluation for every reference tissue and observer were used. A two-way random ICC model, with absolute agreement, single measures and a 95 % confidence interval was used.

## Results

The mean and standard deviation of the rCBV measurements for all observers are shown in Table 1. There was no statistically significant difference between the rCBV measurements of the two evaluations of all tissues, except for observer 1 in NAWM tumour.

**Table 1:** rCBV measurements with different reference tissues by three observers on two occasions

Intra-observer	Observer 1			Observer 2			Observer 3		
reference tissue	1st evaluation	2nd evaluation	*p*	1st evaluation	2nd evaluation	*p*	1st evaluation	2nd evaluation	*p*
NAWM by choice	6.1 ± 2.7	6.1 ± 2.9	0.95	8.2 ± 4.3	8.3 ± 3.3	0.90	8.5 ± 3.3	9.2 ± 3.8	0.14
NAGM by choice	2.7 ± 1.3	2.7 ± 1.3	0.61	3.0 ± 1.7	2.6 ± 1.1	0.06	2.8 ± 1.2	2.6 ± 1.1	0.17
NAWM tumor	5.8 ± 2.8	5.1 ± 2.4	0.02*	7.5 ± 4.2	7.9 ± 3.9	0.22	7.4 ± 4.3	7.8 ± 3.7	0.28
Putamen	3.3 ± 1.4	3.4 ± 1.7	0.26	3.3 ± 1.8	2.9 ± 1.3	0.07	3.1 ± 1.5	2.9 ± 1.3	0.16
Frontal NAWM	6.6 ± 2.8	6.2 ± 2.6	0.33	6.4 ± 3.0	6.7 ± 3.1	0.39	7.6 ± 3.1	7.6 ± 3.1	0.89
Parietal NAWM	7.7 ± 3.8	7.4 ± 4.1	0.71	7.6 ± 3.7	8.5 ± 4.2	0.12	9.0 ± 3.8	8.8 ± 4.0	0.68
Thalamus	3.9 ± 1.9	3.7 ± 1.7	0.49	2.9 ± 1.9	3.1 ± 1.4	0.50	2.7 ± 1.1	2.5 ± 1.2	0.23
Centrum semiovale	9.8 ± 3.9	9.7 ± 3.7	0.62	10.0 ± 4.1	10.7 ± 4.9	0.07	10.0 ± 3.9	10.1 ± 4.0	0.55

*p<0.05 was considered statistically significant.

### Intra-observer variability

**Fig. 2:**
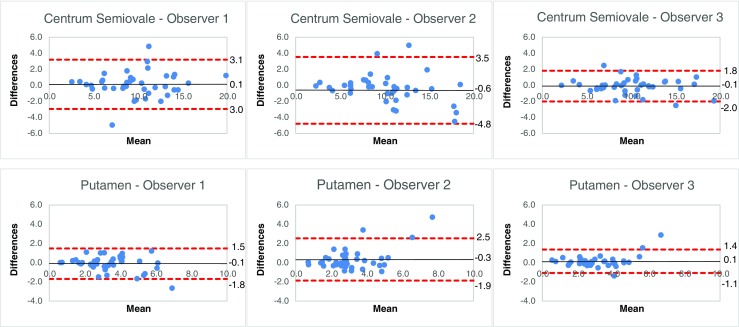
Intra-observer Bland-Altman plots. Bland-Altman plots were used to analyse the agreement between the two evaluations per observer. The difference between two evaluations of one observer was plotted on the vertical axis and the mean of the two evaluations was plotted on the horizontal axis. The solid (black) line represents the mean value for the data points and the dashed (red) line represents the 1.96*SD

**Fig. 3:**
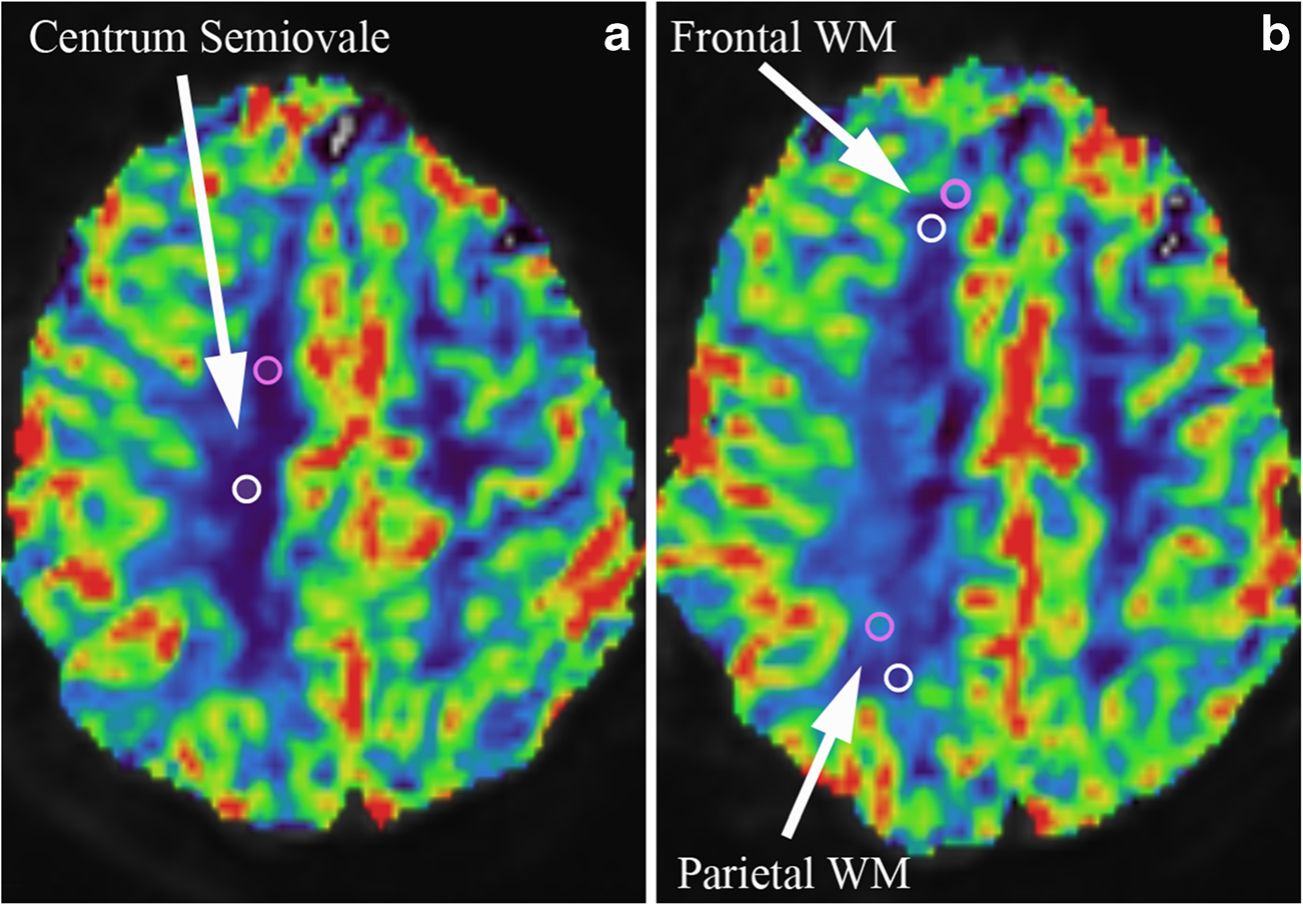
The region of interest (ROI) placement on the rCBV for selecting the reference tissue of centrum semiovale (A), the frontal white matter (WM) (B), and parietal WM (B). The effects of the ROI placement are shown in Table 3

The ICC, CV and Bland-Altman analysis for intra-observer variability are summarized in Table 2. The ICC ranged from 0.50 to 0.97 for all tissues, indicating fair to excellent agreement. The averaged CV for a reference tissue ranged from 5.1 % to 22.1 % for all reference tissues and observers. Centrum semiovale (range ICC 0.88–0.97) was the only reference tissue that showed excellent agreement (ICC >0.74) for all observers. Centrum semiovale showed the lowest averaged CVs for all observers (range 5.1–9.0 %). Results of the Bland-Altman analysis showed that the mean differences for centrum semiovale and putamen were close to zero. Bland-Altman plots of centrum semiovale and putamen for intra-observer variability are shown in Fig. 2. Figure 3 illustrates the effect of ROI placement and the rCBV between centrum semiovale, the frontal NAWM and parietal NAWM. The effects for this example are shown in Table 3. The difference in rCBV values between two ROIs for frontal NAWM is 21 %, and for parietal NAWM 30 % compared to 3 % for centrum semiovale.

**Table 2:** Intra-observer agreement – intraclass correlation coefficient, coefficient of variation, and Bland-Altman analysis

Intra-observer agreement	Observer 1			Observer 2			Observer 3		
rCBV reference tissues	ICC	CV	BA	ICC	CV	BA	ICC	CV	BA
NAWM by choice	0.68 (0.47–0.82)	15.2 ± 16.6	0.0 ± 2.3 (-4.4–4.5)	0.62 (0.38–0.78)	16.2 ± 14.2	-0.1 ± 3.4 (-6.7–6.6)	0.71 (0.51–0.84)	16.6 ± 16.2	-0.7 ± 2.7 (-5.9–4.6)
NAGM by choice	0.67 (0.45–0.81)	16.1 ± 16.6	0.1 ± 1.1 (-2.0–2.2)	0.67 (0.46–0.82)	17.0 ± 13.9	0.4 ± 1.1 (-1.9–2.6)	0.86 (0.74–0.92)	9.1 ± 12.2	0.1 ± 0.6 (-1.1–1.3)
NAWM tumor	0.73 (0.53–0.85)	19.0 ± 14.7	0.7 ± 1.9 (-3.0–4.5)	0.87 (0.77–0.93)	13.0 ± 15.7	-0.4 ± 2.0 (-4.4–3.6)	0.88 (0.78–0.93)	15.3 ± 18.3	-0.3 ± 2.0 (-4.2–3.5)
Putamen	0.86 (0.75–0.93)	11.5 ± 10.0	-0.1 ± 0.8 (-1.8–1.5)	0.73 (0.54–0.85)	14.1 ± 13.8	0.3 ± 1.1 (-1.9–2.5)	0.90 (0.81–0.94)	8.5 ± 9.2	0.1 ± 0.6 (-1.1–1.4)
Frontal NAWM	0.68 (0.46–0.82)	16.6 ± 14.6	0.3 ± 2.2 (-3.9–4.6)	0.72(0.53– 0.84)	19.1 ± 20.4	-0.3 ± 2.3 (-4.8–4.1)	0.87 (0.76–0.93)	10.3 ± 8.8	0.0 ± 1.6 (-3.1–3.2)
Parietal NAWM	0.59 (0.34–0.76)	22.1 ± 21.5	0.2 ± 3.6 (-6.8–7.3)	0.61 (0.38–0.78)	21.7 ± 20.5	-0.9 ± 3.4 (-7.6–5.9)	0.70 (0.50,0.83)	13.1 ± 14.5	0.2 ± 3.1 (-5.8–6.2)
Thalamus	0.50 (0.22–0.70)	21.1 ± 18.1	0.2 ± 1.8 (-3.3–3.7)	0.53 (0.26–0.72)	17.5 ± 18.5	-0.2 ± 1.6 (-3.3–3.0)	0.73 (0.54–0.85)	16.8 ± 16.0	0.2 ± 0.8 (-1.5–1.8)
Centrum semiovale	0.92 (0.85–0.96)	8.2 ± 9.2	0.1 ± 1.6 (-3.0–3.1)	0.88 (0.79–0.94)	9.0 ± 8.9	-0.6 ± 2.1 (-4.8–3.5)	0.97 (0.94–0.98)	5.1 ± 5.4	-0.1 ± 1.0 (-2.0–1.8)

*ICC* intraclass correlation coefficient. Numbers in parentheses are 95 % confidence intervals

*CV* coefficient of variation. Data are mean CV ± standard deviations

*BA* Bland-Altman analysis. The mean difference, standard deviation and in parentheses 95 % limits of agreement are shown

*NAWM* normal-appearing white matter

**Table 3:** Example of effects of two ROI placements in white matter

Location	Evaluation time	CBV	rCBV	% rCBV_t1-t2_
Centrum semiovale	1. purple	0.34 ± 0.050	6.96	100 %
	2. white	0.33 ± 0.012	7.17	103 %
Frontal WM	1. purple	0.49 ± 0.057	4.88	100 %
	2. white	0.62 ± 0.158	3.85	79 %
Parietal WM	1. purple	0.46 ± 0.057	5.18	100 %
	2. white	0.66 ± 0.068	3.62	70 %

Table 3 shows the effects of region of interest (ROI) placement in white matter (WM) for the example in Fig. 2. The difference in rCBV values between two ROIs for frontal WM is 21 %, and for parietal WM 30 % difference compared to 3 % for centrum semiovale

### Inter-observer variability

**Fig. 4:**
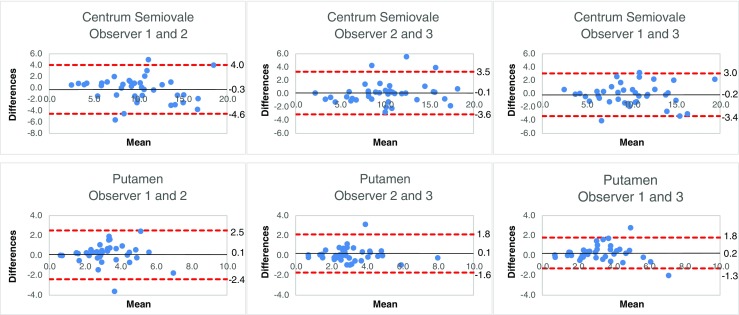
Interobserver Bland-Altman plots. Bland-Altman plots were used to analyse the agreement between two observers. The difference between the first measurement of two observers was plotted on the y-axis and the mean of the two evaluations was plotted on the x-axis. The solid (black) line represents the mean value for the data points and the dashed (red) line represents the 1.96*SD

The ICC, CV and Bland-Altman analysis for inter-observer variability are summarized in Table 4. The ICC for al pairwise observer combinations ranged from 0.44 to 0.92 for all tissues, indicating poor to excellent agreement. The averaged CV for a reference tissue ranged from 8.1 % to 31.1 % for all reference tissues and the pairwise combination of observers. Centrum semiovale (range ICC 0.88–0.97) was the only reference tissue that showed excellent agreement (ICC >0.74) for all pairwise combination of observers. Centrum semiovale (range 8.1–12.5 %) showed the lowest averaged CVs. Results of the Bland-Altman analysis showed that the mean differences for centrum semiovale and putamen were close to zero. Bland-Altman plots of centrum semiovale and putamen for interbserver variability are shown in Fig. 4.

**Table 4:** Interobserver agreement – intraclass correlation coefficient, coefficient of variation, and Bland-Altman analysis

Inter-observer agreement	Observer 1 vs. Observer 2			Observer 2 vs. Observer 3			Observer 1 vs. Observer 3		
rCBV Reference Tissues	ICC	CV	BA	ICC	CV	BA	ICC	CV	BA
NAWM by choice	0.44 (0.12–0.68)	26.3 ± 18.1	-2.1 ± 3.6 (-9.1–4.9)	0.52 (0.24–0.72)	17.5 ± 16.8	-0.4 ± 3.8 (-7.8–7.1)	0.44 (0.01–0.71)	27.7 ± 21.0	-2.4 ± 2.8 (-7.8–3.0)
NAGM by choice	0.71 (0.51–0.84)	14.9 ± 14.3	-0.2 ± 1.1 (-2.5–2.0)	0.62 (0.38–0.78)	19.7 ± 15.4	0.1 ± 1.3 (-2.4–2.7)	0.66 (0.44–0.81)	17.6 ± 14.8	0.0 ± 1.0 (-2.0–2.0)
NAWM tumour	0.73 (0.37–0.88)	23.5 ± 16.8	-1.6 ± 2.3 (-6.2–2.9)	0.85 (0.73–0.92)	18.8 ± 19.1	0.1 ± 2.3 (-4.3–4.5)	0.70 (0.40–0.85)	23.9 ± 18.6	-1.6 ± 2.6 (-6.7–3.5)
Putamen	0.71 (0.51–0.84)	15.1 ± 14.6	0.1 ± 1.3 (-2.4–2.5)	0.82 (0.69–0.90)	12.1 ± 11.5	0.1 ± 0.9 (-1.6–1.8)	0.85 (0.72–0.92)	11.5 ± 9.9	0.2 ± 0.8 (-1.3–1.8)
Frontal NAWM	0.77 (0.61–0.88)	17.7 ± 16.1	0.2 ± 2.0 (-3.7–4.0)	0.72 (0.53–0.84)	19.1 ± 20.3	-1.2 ± 1.8 (-4.7–2.3)	0.75 (0.49–0.87)	15.8 ± 13.7	-1.0 ± 1.9 (-4.8–2.7)
Parietal NAWM	0.69 (0.48–0.82)	20.7 ± 17.2	0.1 ± 3.0 (-5.8–5.9)	0.50 (0.23–0.70)	26.4 ± 21.9	-1.4 ± 3.7 (-8.5–5.8)	0.51 (0.24–0.71)	26.1 ± 19.4	-1.3 ± 3.7 (-8.6–6.0)
Thalamus	0.31 (0.02–0.56)	31.1 ± 22.1	1.0 ± 2.2 (-3.4–5.3)	0.53 (0.26–0.72)	19.1 ± 20.3	0.1 ± 1.4 (-2.7–2.9)	0.49 (0.05,0.74)	25.1 ± 17.2	1.2 ± 1.4 (-1.5–3.8)
Centrum semiovale	0.85 (0.73–0.92)	12.5 ± 10.3	-0.3 ± 2.2 (-4.6–4.0)	0.92 (0.85–0.96)	8.1 ± 8.4	-0.1 ± 1.8 (-3.6–3.5)	0.91 (0.84–0.95)	9.1 ± 8.2	-0.2 ± 1.6 (-3.4–3.0)

*ICC* intraclass correlation coefficient. Numbers in parentheses are 95 % confidence intervals

*CV* coefficient of variation. Data are mean CV ± standard deviations

*BA* Bland-Altman analysis. The mean difference, standard deviation and in parentheses 95 % limits of agreement are shown

*NAWM* normal-appearing white matter

## Discussion

Several factors may influence rCBV values [24], including contrast agent characteristics, acquisition technique and data pre- and post-processing. A low variability of rCBV measurements is not only important for accurate tumour grading and treatment monitoring, it also enables comparisons of values across studies and patient populations.

In this study we have shown that selecting the contralateral centrum semiovale as reference tissue for rCBV measurements in DSC-MRI of glioma patients provides the lowest intra- and inter-observer variability. We assessed the observers’ variability of reference tissue selection. In total eight regions of interest were depicted as reference tissue in NAWM and NAGM. Overall, a wide variability in observer agreement of the rCBV measurements was reported in our study.

The centrum semiovale is easier to annotate compared to the frontal or parietal NAWM, areas which are hindered by partial volume effects of WM and GM, pronounced T2-shortening effects of the cortical vessels (mainly GM) and distortion artifacts due to the frontal sinus. The centrum semiovale is a large homogenous area of WM that is mostly visible in only one or two axial slices, and suffer less from the problems described for the frontal or parietal NAWM. This explains the excellent intraclass correlation coefficient and the low coefficient of variation.

The putamen is a well-defined homogenous area of subcortical GM and could potentially also be a good reference tissue. Thalamus is less suited as a reference tissue because it is more heterogeneous with nuclei and suffers from pronounced T2-shortening effects from vessels in the near vicinity. Putamen showed good to excellent intra- and inter-observer agreement, and showed the lowest averaged CVs for all observers of the GM reference tissues. However, centrum semiovale ICC and CV for intra- and inter-observer agreement were slightly better compared to putamen.

**Fig. 5:**
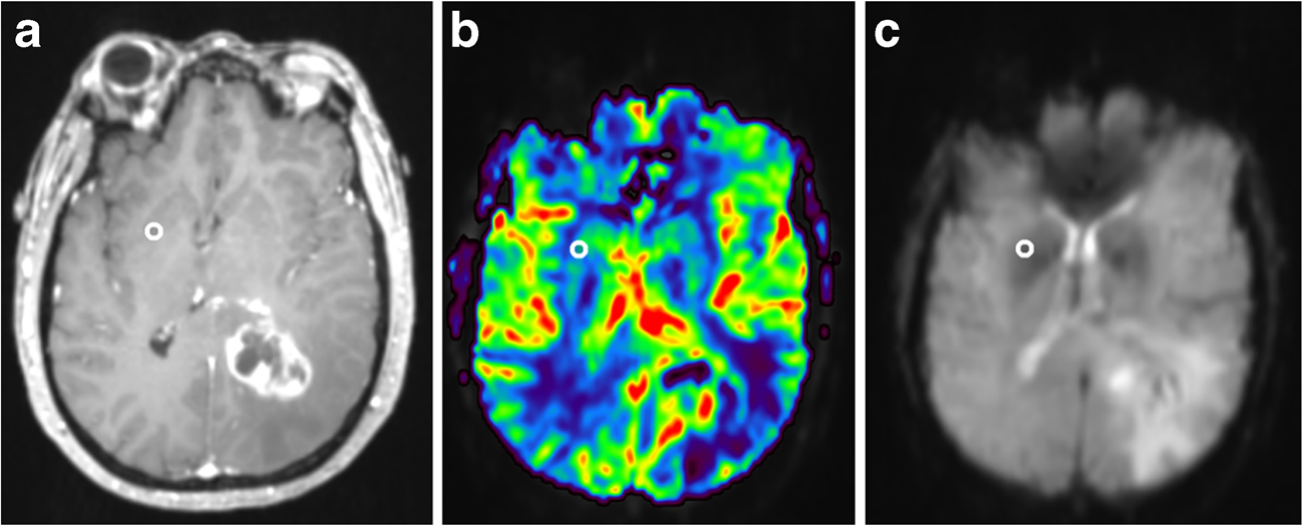
Example of distortion artifacts in the putamen in a patient with a glioblastoma in the left hemisphere (A contrast-enhanced T1-weighted MR image, B cerebral blood volume (CBV) map from dynamic susceptibility contrast-enhanced MRI (DSC-MRI), C source DSC-MR images). Note that the CBV map is calculated, also in the putamen area. However, DSC-MR source images show distortion artifacts in the putamen area. Despite these distortions rCBV could be calculated in the putamen area

Our data showed distortion artifacts due to the GE-EPI sequence, which is common near brain-bone-air interfaces, mainly in the frontal lobe and in the area of the putamen [24]. Despite these distortions, rCBV in putamen could still be calculated (see Fig. 5). Distortions in these areas can be decreased by changing the phase encoding order from posterior to anterior instead of anterior to posterior. Further research on the reproducibility of the DSC-MR images over time is needed to investigate the distortion artifacts near the putamen and to investigate the effect of distortions on the calculation of rCBV.

Besides artifacts (like distortion artifacts and pronounced T2-shortening effects), insufficient Z-coverage can be a problem in DSC-MRI (which was not the case in our study) and centrum semiovale can be excluded from the scan. If centrum semiovale and putamen are not available for assessment then normal-appearing white matter in the slice of the tumour also showed good ICC for intra- and inter-observer variability. However, it also showed an average CV of >20 % for inter-observer variability, which is not preferable. Since artifacts are the most common occurrences to hinder normalization we advise selecting normal-appearing white matter far away from the sinuses and mastoid to avoid distortion artifacts and to stay away from vessels to avoid pronounced T2-shortening effects.

Only one related work was found that assessed the observer agreement of selecting reference tissues. Wetzel et al. [18] investigated the observer agreement of the tumour ROI and used one pixel in NAWM as reference tissue of which the exact location was not described. To analyse the precision of measurements of NAWM they selected ten ROIs close to the initial reference ROI in NAWM and showed a CV of 20 % of repeated measurements in NAWM. In our study, if NAWM is selected as reference tissue then the results showed a higher overall CV, from 23.9 % for NAWM in the slice of the tumour, 26.4 % for parietal NAWM up to 27.7 % for NAWM by choice, except for the centrum semiovale, which showed the lowest CV (range 8.1–12.5 %). Another explanation for the differences in CV (besides partial volume effects of WM and GM, pronounced T2-shortening effects or distortion artifacts) could be the size of the ROI. The use of only one pixel as reference tissue by Wetzel et al. [18] is a limitation and can explain the higher CV in their study compared to our CV for centrum semiovale since centrum semiovale is an easy to annotate homogenous area and not hindered by the problems described above. The size of the ROI is still a matter of debate, and ranges in the literature from 3.2 mm^2^ [18] to 50 mm^2^ [3], and even up to 432 mm^2^ [1]. That is, size ranged from 1 pixel [18] up to 100 pixels [1]. We decided to use 25 mm^2^ ROIs since these ROIs can be easily placed in cortical GM, but also in thalamus and putamen without partial volume averaging within the NAWM.

We chose to use circular ROIs with a fixed diameter instead of freehand ROIs for the reference tissue. In a previous preliminary study [25], we showed that the freehand ROIs were larger than the ROIs used in the current study and therefore showed lower CV. However, freehand ROIs showed lower agreement (lower ICC), because it is difficult to draw the same freehand ROI twice or by different observers. We therefore recommended using ROIs with fixed diameters.

Our goal was to only assess the observer agreement when selecting the reference tissue, and therefore the tumour hotspot was a fixed tumour throughout the experiments. Based on our study we cannot assess the overall influence if the observers were allowed to choose both the tumour hotspot and the reference tissue. Wetzel et al. [18] showed that the inter-observer CV for determining the tumour hotspot ROI with a fixed reference tissue ROI is 30 %. Our study showed that the CV for the reference tissue with a fixed tumour hotspot ROI ranged from 8.3 % to 31.1 %. A study should be performed to assess the overall influence if both can freely be selected. An accepted target for measurement error in multicentre studies is a CV that is less than 20 %, according to the Quantitative Imaging Biomarkers Alliance (QIBA) [26]. Our study showed a lower overall inter-observer agreement than intra-observer agreement in the rCBV measurements, which is in concordance to other studies [27–32]. However, these variabilities are difficult to compare to our results because different tumours, body parts, modalities, methods of dynamic acquisitions and pharmacokinetic models are used.

## Limitations

One limitation of our study is that we did not use vessel segmentation in the analyses. It is known that GE-EPI sequences are more weighted towards the macrovasculature. Large vessels are pronounced due to T2 shortening outside the vessel lumen, which results in an overestimation of rCBV in cortical gray matter and nearby white matter [12]. To minimize these macrovessel signals in gradient echo images vessel segmentation techniques can be used during post-processing [33].

Another limitation is that our results only apply to rCBV. Care must be taken to extrapolate the results to other perfusion parameters like cerebral blood flow, spin-echo acquisitions or other perfusion methods (like arterial spin labeling or T1-dynamic contrast-enhanced MR perfusion).

## Conclusion

Our findings show that the observer variability of rCBV measurements can vary between poor and excellent, depending on the chosen reference tissue in NAWM or NAGM. Contralateral centrum semiovale as the internal reference standard for rCBV showed the lowest observer variability.

## Abbreviations

(NA)GM: (Normal-appearing) grey matter
(NA)WM: (Normal-appearing) white matter
(r)CBV: (relative) Cerebral blood volume
CV: Coefficient of variation
DSC-MRI: T2*-weighted dynamic susceptibility contrast-enhanced MR imaging
GE-EPI: Gradient echo-echo planar imaging
ICC: Intraclass correlation coefficient
ROI: Region of interest
T1w/T2w: T1-weighted/T2-weighted
